# The alignment of respiration to sensory-motor events is shaped by expected effort

**DOI:** 10.1016/j.isci.2026.115046

**Published:** 2026-02-17

**Authors:** Christoph Kayser, Lena Hehemann, Lisa Stetza

**Affiliations:** 1 Department for Cognitive Neuroscience, Faculty of Biology, Bielefeld University, Bielefeld, Germany

**Keywords:** neuroscience, behavioral neuroscience, cognitive neuroscience

## Abstract

Humans often align their respiration with external events, which may optimize neural resources for perception and action. This may adjust neurophysiological processes related to neural excitation, attention, or arousal to optimize task performance. However, it remains unclear whether this alignment is a passive entrainment to a task’s overall rhythm or an active process selectively aligning respiration more to highly demanding events. We tested this by recording respiration during three visual discrimination experiments that manipulated the importance of individual trials by imposing response deadlines or manipulating trial value and difficulty. We found that participants align their respiration more consistently for trials with short deadlines or trials presenting high-value and high-difficulty. This demonstrates that respiratory alignment is dynamically modulated on a trial-by-trial basis according to anticipated effort or task demands. Hence, respiration serves as an active tool to strategically allocate cognitive resources for sensory-motor challenges.

## Highlights

• Humans tend to align their respiration with external events
• How actively and selectively they do this remains unclear
• We find that this occurs particularly for high-value and or high-difficulty situations
• Respiration strategically allocates cognitive resources for upcoming challenges

## Graphical abstract

**Figure:**
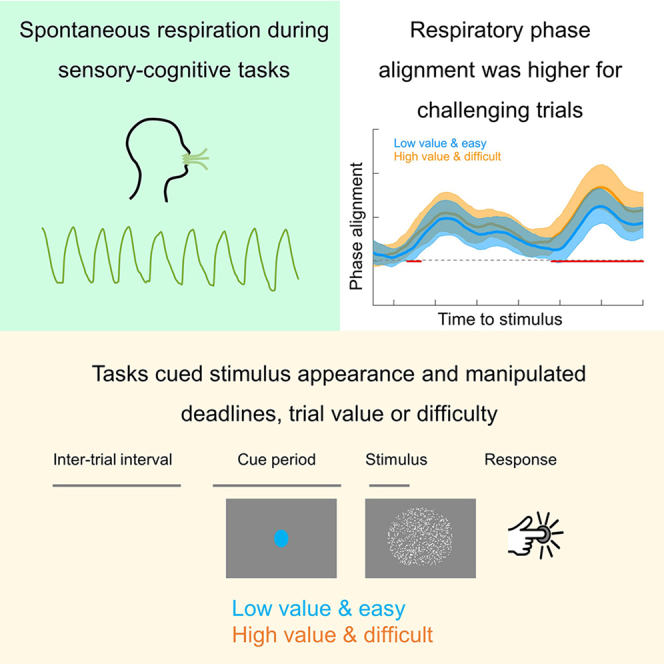

## Introduction

When preparing to perform specific actions or to act upon expected stimuli, we often tend to temporally align our respiration with these events. In fact, the active regulation of respiration is fundamental in some sports or activities demanding precise manual coordination.^1^ However, recent studies suggest that also during seemingly simple laboratory tasks probing sensation or cognition participants tend to structure their respiratory pattern around experimental events like stimulus presentation and response time.^2^^,^^3^^,^^4^^,^^5^^,^^6^ This respiratory alignment explains some of the trial-to-trial variability in response accuracy and reaction times, suggesting that behavioral performance systematically comodulates with respiration.^4^^,^^7^^,^^8^^,^^9^^,^^10^^,^^11^^,^^12^ This alignment of respiration may not only serve to control body movements, e.g., during shooting, but also to guide neural resources, such as excitability or arousal, to ensure the optimal processing of relevant sensory-motor contingencies, in line with theories of active sensing.^13^^,^^14^^,^^15^

Following this notion, recent studies have delineated the relation between respiration and brain activity in detail.^16^^,^^17^^,^^18^^,^^19^^,^^20^ The brain structures controlling respiration and those sensing the resulting changes in airflow or chest pressure are intricately connected with the limbic system.^21^^,^^22^^,^^23^ As a result, direct neural feedback about the respiratory state is widely available in the brain.^24^^,^^25^ Critically, both aperiodic signatures of neural excitability as well as rhythmic signatures of arousal seem to comodulate with the respiratory phase^26^^,^^27^ and respiration directly modulates the encoding of task-specific sensory signals in relevant brain regions.^7^^,^^11^^,^^28^^,^^29^ This suggests that by actively aligning respiration with expected stimuli or actions our brain (consciously or unconsciously) ensures an optimal neural substrate to process or perform these.

Building on these findings, we previously investigated the relation between respiration and task performance across multiple datasets comprising perceptual and cognitive tasks.^2^^,^^5^ Specifically, we studied how consistent the respiratory phase is across trials within individual participants, and we probed how predictive the trial-wise respiratory phase and the trial-average phase are for participants behavior. We found that both the specific respiratory phase preceding a trial and the trial-averaged respiratory phase of individual participants are predictive for their behavioral outcomes: participants who align their respiration “optimally” to the paradigm tended to respond faster than those who align “non-optimally.”^5^ If respiration indeed functions as a mechanism to align neural resources to important events, one could assume that this alignment of respiration is particularly strong when events are expected to place higher demands or to require high effort to be completed successfully. Under the assumption that the nervous system attributes its limited resources optimally,^30^^,^^31^ one would hence expect stronger alignment to more important or effort-demanding events and a reduced alignment to less important or less effort-demanding events.

**Figure 1:**
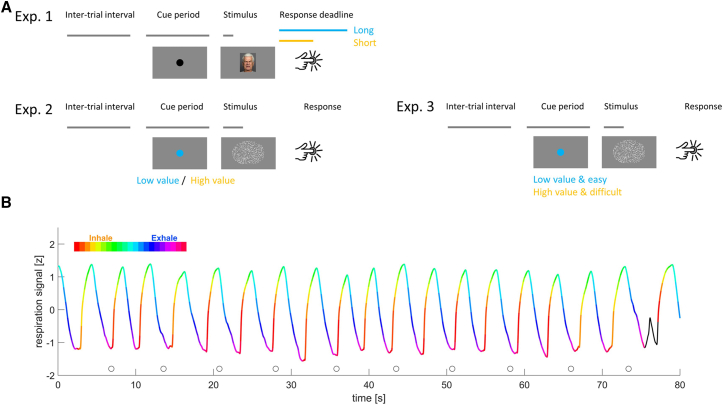
Paradigms and example data (A) Schematic of the three experiments. Each experimented featured pseudo-random inter-trial intervals, a fixed cueing period indicating the upcoming stimulus, a stimulus, and a response period. Experiment 1 featured an emotion discrimination task and contrasted conditions with long and short response deadlines presented in different blocks. Experiments 2 and 3 featured a random dot motion task and manipulated trial value and difficulty. Experiment 2 manipulated both independently, while experimented 3 yoked them. Trial value was indicated by the cue. (B) Example respiratory trace from one participant. Color indicates the respiratory phase, inhalation is shown upwards. Circles indicate stimulus onset times. The black part of the curve is an example of an atypical cycle.

Yet, most previous studies on respiratory task alignment only focused on the overall alignment of respiration regardless of task demands and did not explicitly manipulate the incentive to optimize the alignment of respiration to specific individual trials in a flexible manner by manipulating the importance of individual trials.^2^^,^^3^^,^^4^^,^^5^^,^^6^ Hence it remains unclear whether the hypothesis of a differential alignment to more and less important events indeed holds true. We here test this hypothesis based on three perceptual tasks in which we manipulated importance of individual trials, operationalized either by imposing loose or tight response deadlines, or by the explicit manipulation of trial value and difficulty in an unpredictable manner across trials (Figure 1). Overall, our data support that participants align their respiration more reliably to events imposing higher demands or holding higher importance.

## Results

We tested the active alignment of respiration to the sequence of trials in three experiments manipulating either the requirements on response speed (experiment 1) or the importance of submitting a correct response by manipulating the value of individual trials on a trial-by-trial basis (experiments 2 and 3; see Figure 1). We designed these experiments based on previous work demonstrating the overall tendency of participants to align their respiration to experimental trials but introduced systematic manipulations of response deadlines, trial-value, and difficulty. The time intervals between subsequent stimulus onsets were on the order of 5.7–7.5 s. This timescale is longer than the typical respiratory cycle duration for most participants and hence should be compatible with an active alignment of respiration to the experimental sequence. For the present data, the average durations of respiratory cycles across three experiments were 3.79 ± 0.12 s (mean ± s.e.m., *n* = 27 participants) for experiment 1, 4.23 ± 0.23 s (*n* = 24 participants) for experiment 2 and 3.55 ± 0.11 s for experiments 3 (*n* = 23 participants), respectively.

### Influence of response deadline

**Figure 2:**
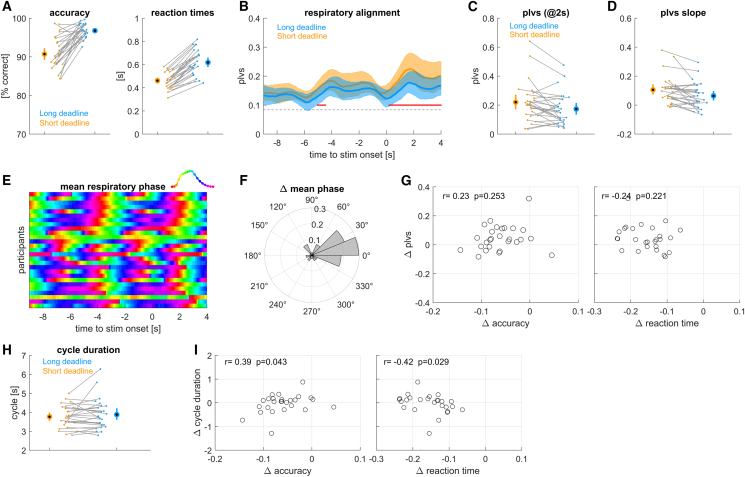
Results from experiment 1 manipulating response deadlines (A) Accuracy and reaction times for short and long response deadlines (see color code). (B) Alignment of respiration to stimulus onset, measured using the phase-locking vector strength (plvs). Red lines indicate significant differences (cluster-based permutation test, at *p* < 0.01). (C) Participant- and condition-wise plvs values at +2 s. (D) Slope of plvs change around stimulus onset. (E) Trial-averaged respiratory phase for each participant, greenish colors indicate peak inhalation. (F) Condition difference in trial-averaged respiratory phase shown as angular histogram. (G) Participant-wise condition-differences in plvs vs. the differences in response accuracy and reaction times. r- and *p* values are based on a Spearman rank correlation. (H) Average duration of those respiratory cycles including stimulus onset. (I) Participant-wise differences in respiratory cycle duration vs. differences in response accuracy and reaction times. Group-level data are shown as mean and 95% percentile bootstrap confidence intervals, dots, and thin lines indicate single participant data. *N* = 27 participants.

We first asked how changes in the demands on response speed affect the respiratory alignment to the experimental trials. In experiment 1, we contrasted trials performed with a LONG reaction time deadline (1.4 s) to trials performed on an SHORT deadline performed in different blocks (tailored to the 30^th^ percentile of RTs observed during the LONG condition). As expected, reaction times were significantly shorter in the SHORT compared to the LONG condition (Figure 2A; 0.461 ± 0.013 s vs. 0.618 ± 0.021 s, paired *t* test *p* < 10^−5^, *t* = 16.17, Cohen’s *D* = 3.17; the effect is visible in the figure by the majority of single participant data points pointing in the same direction; *n* = 27 participants) and response accuracy was significantly lower in the SHORT condition (90.7 ± 1% vs. 96.8 ± 0.3% correct responses, *p* < 10^−5^, *t* = 7.77, Cohen’s *D* = 1.52).

The within-participant alignment of the respiratory phase was higher around the stimulus-response times compared to the period a few seconds earlier. This phase locking was significantly stronger than expected by chance (permutation test against surrogate data; *p* < 0.01 corrected for multiple tests a long time). More importantly, this phase locking was significantly stronger in the SHORT compared to the LONG condition (Figure 2B). A cluster-based permutation test correcting for multiple comparisons over time revealed two significant clusters (cluster 1: −5.05 to −4.45 s, *p* = 0.004, Cohen’s *D* = 0.38; cluster 2: 0.55–4.00 s, *p* = 0.004, Cohen’s *D* = 0.55). Figure 2C illustrates the participant wise plvs values for both conditions at *t* = 2 s. To further characterize this dynamic alignment of respiration, we computed the slope of the plvs around each stimulus onset (Figure 2D). This is intended to capture how quickly respiratory alignment adjusts to the expected stimulus onset and was significantly higher in the SHORT compared to the LONG condition (*p* = 0.0018, *t* = 3.48, Cohen’s *D* = 0.67). Hence, our data show that participants tend to align their respiratory phase to the expected upcoming trials significantly tighter in the SHORT condition.

To probe how similar or different individual participants tended to breathe around trials, we compared the trial-averaged respiratory phases between participants (Figure 2E). Visibly, this average phase was comparable across participants and most participants were at peak inhalation around stimulus onsets. Across participants, the trial-average respiratory phase was also similar between SHORT and LONG conditions (Figure 2F; randomization test comparing the mean angles, mean difference 0.145 radians, 95% percentile CI: [−0.395, 0.397], *p* = 0.483).

We also asked whether the condition difference in the behavioral data relates to the condition difference in plvs. For this, we computed the correlation of the condition difference in plvs to the respective differences in reaction times and accuracy across participants. These correlations were not significant (Figure 2G; see Spearman rank correlations indicated there).

Finally, we probed whether the durations of participants respiratory cycles differed between the two conditions. For this, we extracted for each trial the duration of that respiratory cycle including stimulus onset. These durations did not differ significantly between conditions (Figure 2H; paired *t* test, *p* = 0.310, *t* = 1.04, Cohen’s *D* = 0.199). However, the within participant condition differences did correlate significantly with the condition effects on response accuracy (Figure 2I; r = 0.39, *p* = 0.043) and for reaction times (r = −0.42, *p* = 0.029). The data suggest that those participants whose behavior was least affected by the SHORT reaction time deadline were those who exhibited comparable respiratory cycle durations in both conditions or who had longer respiratory cycles during the SHORT condition.

### Influence of trial value independent of difficulty

In a second experiment, we manipulated the trial-wise value of correct responses and task difficulty as independent factors. The trial-wise value was cued by a 3-s cue preceding stimulus onset whereas task difficulty was not cued in advance and could only be inferred during stimulus presentation, thus remaining unpredictable. We expected that participants would structure their respiratory phase more systematically around high compared to low value trials, and that they would perform better in these high-value trials. We did not expect an influence of trial difficulty on respiration, since difficulty was not cued. Yet, we did expect participants to perform better during easier trials.

**Figure 3:**
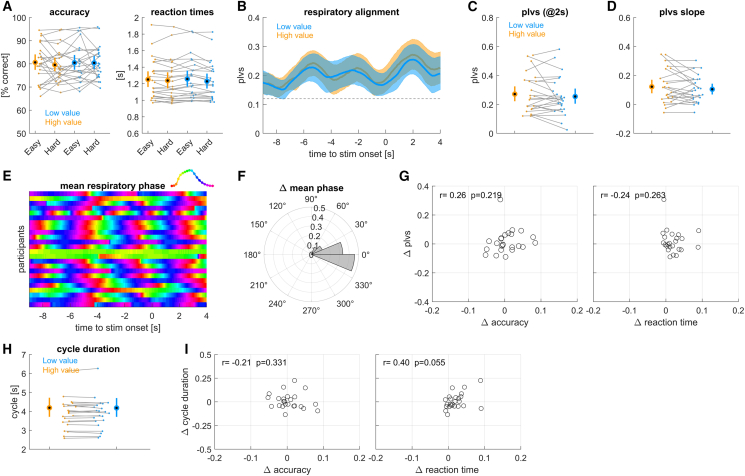
Results from experiment 2 manipulating trial value and difficulty independently (A) Accuracy and reaction times for each condition. High value trials are orange, low value trials blue. Behavioral data are additionally split by difficulty (easy and hard). (B) Alignment of respiration to stimulus onset, measured using the phase-locking vector strength (plvs) for each value, averaged over difficulty levels. (C) Participant- and condition-wise plvs values at +2 s. (D) Slope of plvs change around stimulus onset. (E) Trial-averaged respiratory phase for each participant, greenish colors indicate peak inhalation. (F) Condition difference in trial-averaged respiratory phase shown as angular histogram. (G) Participant-wise condition-differences in plvs vs. the differences in response accuracy and reaction times. r- and *p* values are based on a Spearman rank correlation. (H) Average duration of those respiratory cycles including stimulus onset. (I) Participant-wise differences in respiratory cycle duration vs. differences in response accuracy and reaction times. Group-level data are shown as mean and 95% percentile bootstrap confidence intervals, dots and thin lines indicate single participant data. *N* = 24 participants.

The behavioral data revealed small effects of value and difficulty. For response accuracy neither effect was significant (Figure 3A; value: *F* = 0.62, *p* = 0.433; difficulty: *F* = 0.24, *p* = 0.628; *n* = 24 participants) and the difference between high hand low value trials was small (high value: 80.5 ± 1.5%, low value: 80.0 ± 1.4%). For reaction times, the effect of value was significant (*F* = 11.4, *p* = 0.001), while that of difficulty was not (*F* = 0.07, *p* = 0.794). Reaction times were longer for high value trials (high value: 1.254 ± 0.05 s, low value: 1.233 ± 0.05 s). Hence the manipulation of value had a significant but numerically small effect on behavior. This let us to expect that any effect of value on respiratory alignment would also be small.

Indeed, while the overall alignment of respiration to the experimental paradigm was significant, the plvs did not differ between high and low value conditions (Figure 3B; a cluster-based permutation test did not reveal any significant clusters at *p* < 0.05). The slope of the plvs also did not differ significantly (Figure 3D, paired *t* tests, *p* = 0.286, *t* = 1.09, Cohen’s *D* = 0.223). As for experiment 1, participants tended to reach peak inhalation at the time of stimulus onset (Figure 3E) and the trial-averaged respiratory phase did not differ between value conditions (angular difference: mean = 0.106 radians, 95% CI: [−0.143, 0.145], *p* = 0.160; Figure 3F). The correlations between condition differences in behavior and respiration were not significant (Figures 3G and 3I; see correlation values there) and the duration of respiratory cycles was also comparable between conditions (Figure 3H; *p* = 0.602, *t* = 0.53, Cohen’s *D* = 0.108).

### Influence of linked trial value and difficulty

**Figure 4:**
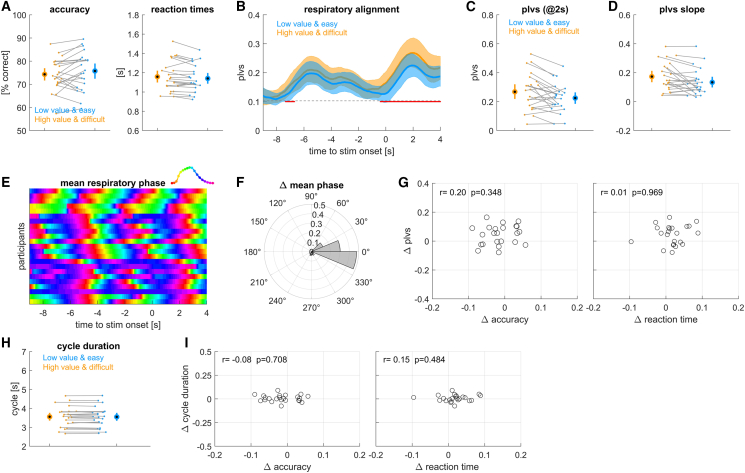
Results from experiment 3 linking trial value and difficulty (A) Accuracy and reaction times for high value and difficult trials (orange) and low value and easy trials (blue). (B) Alignment of respiration to stimulus onset, measured using the phase-locking vector strength (plvs). Red lines indicate significant differences (cluster-based permutation test, at *p* < 0.01). (C) Participant- and condition-wise plvs values at +2 s. (D) Slope of plvs change around stimulus onset. (E) Trial-averaged respiratory phase for each participant, greenish colors indicate peak inhalation. (F) Condition difference in trial-averaged respiratory phase shown as angular histogram. (G) Participant-wise condition-differences in plvs vs. the differences in response accuracy and reaction times. r- and *p* values are based on a Spearman rank correlation. (H) Average duration of those respiratory cycles including stimulus onset. (I) Participant-wise differences in respiratory cycle duration vs. differences in response accuracy and reaction times. Group-level data are shown as mean and 95% percentile bootstrap confidence intervals, dots and thin lines indicate single participant data. *N* = 23 participants.

The lack of effects in experiment 2 may have resulted from an overall low effectiveness of the manipulation of value, and or the fact that high and low value tasks had on average comparable difficulty, and hence possibly required similar efforts. We hence repeated this experiment but this time linked task difficulty and value, making the high value trials more difficult compared to the low value trials. As a result, both dimensions were known by the cue prior to each stimulus. This combined manipulation of value and difficulty resulted in a significant difference in reaction times between conditions, with reaction times in high value trials being longer compared to low value trials (Figure 4A; high value: 1.158 ± 0.033 s; low value: 1.140 ± 0.030; *p* = 0.040, *t* = 2.18, Cohen’s *D* = 0.46; *n* = 23 participants). Response accuracy did not differ significantly between conditions (high value: 74.3 ± 1.4%, low value: 75.7 ± 1.7%; *p* = 0.110, *t* = 1.66, Cohen’s *D* = 0.35).

The within-participant phase locking of respiration was significant, and importantly, was significantly stronger in the high-compared to the low value condition (cluster-based permutation test revealed a significant cluster from −7.35 to −6.7 s, *p* = 0.004, Cohen’s *D* = 0.49 and one from −0.40 to 4.0 s, *p* = 0.004, Cohens’ *D* = 0.71; Figure 4B and the individual data in Figure 4C). Furthermore, this difference in plvs was accompanied by a difference on phase locking dynamics, and the slope of plvs was significantly higher in the high value condition (Figure 4D; *p* = 0.008, *t* = 2.494, Cohen’ *D* = 0.61). Similar to the other experiments, the trial-averaged phase angles did not differ systematically across participants (Figures 4E and 4F; angular difference: mean = −0.11 radians, 95% CI: [−0.187, 0.181], *p* = 0.310) and the condition-differences in plvs did not correlate significantly with the respective differences in accuracy or reaction times (Figure 4G). The durations of respiratory cycles did not differ significantly between conditions (*p* = 0.379, *t* = 0.90, Cohen’s *D* = 0.187; Figure 4H) and these condition differences in respiratory cycle duration did not correlate with differences in accuracy or reaction times (Figure 4I).

## Discussion

Previous studies have shown that participants tend to align their respiratory cycles to expected but possibly pseudo-randomly timed experimental trials.^2^^,^^3^^,^^4^^,^^5^^,^^6^ Based on the general assumption that respiration is actively structured to support skilled actions or optimize perception, this alignment would confer a specific advantage for behavior and perception, as it sets the brain to an optimal state to process each stimulus. Alternatively, such alignment could also reflect synchronization to the overall experimental pace rather than to trial-specific demands. If respiration was merely entrained to the overall pace of the task one would expect only a general alignment to experimental trials, without selective enhancement on trials carrying particular behavioral relevance. To test for such selective alignment of respiration, we increased behavioral relevance of trials by manipulating the task-related effort through either shortening the response deadlines or varying trial value and difficulty in a pseudo random manner across trials. Overall, our data support that participants tend to breathe more consistently on trials requiring more effort to respond accurately and hence support respiration as an active tool to guide neural resources to challenging sensory-motor events.^14^^,^^15^^,^^16^^,^^17^

It is known that humans tend to breathe in a structured manner around expected events, such as during sports, during conversation, and also in a laboratory task.^1^^,^^2^^,^^3^^,^^32^^,^^33^ This alignment of respiration to such events manifests in a consistency of the respiratory phase across trials. In previous experiments, the timing of experiment trials was often pseudo-random, as is common for neuro-cognitive laboratory tasks. This temporal uncertainty is usually introduced to avoid effects of precise temporal expectation, which can act as a confounder depending on the experimental question.^34^^,^^35^^,^^36^ Despite this temporal uncertainty participants tend to structure their respiration around the experimental events, which typically unfold on a timescale of several seconds, a scale similar to that of individual respiratory cycles. Hence, it could be that in these previous studies participants aligned their respiration to the overall timescale of the experiment, rather than to the individual stimuli or motor actions. Such an entrainment of respiration to the pacing of the experiment would not predict selective differences in the alignment to individual trials but only suggest that the respiratory cycle is timed relative to the experimental timing significantly more than expected by chance.

Yet, the notion that the brain optimally allocates neural resources predicts that these resources should be particularly deployed for important events, i.e., those that require greater effort or which otherwise hold particular value. Recent studies suggest respiration serves to structure neurophysiological processes related to attention, arousal, and neural excitability.^16^^,^^21^^,^^25^^,^^26^^,^^27^^,^^28^ Hence, specifically structuring respiration around critical events would set the brain into a state to optimally react to these. Our data support this hypothesis based on two experimental manipulations: one based on the combined manipulation of difficulty and value of individual trials, and one based on a manipulation of response deadlines. The latter required participants to exploit the sensory information more swiftly to respond, and punished late responses by not letting participants respond to this trial anymore. Since the manipulation of deadlines was imposed in blocks of trials, this result by itself only shows an influence of task demands on alignment but does not prove the selective and trial-by-trial flexible alignment of respiration. The latter, however, was confirmed in experiment 3, which manipulated value and difficulty randomly from trial to trial and where participants could earn more points on difficult compared to easy trials. The results from experiment 3 (but not experiment 2) directly corroborate the selective and trial-wise alignment of respiration, which speaks against the notion of an overall entrainment of respiration to the pacing of the experimental paradigm.

Yet, such a selective alignment does not contradict the notion that some form of temporal entrainment contributes to the alignment of respiration. In most studies on respiratory alignment the time scales of relevant events and respiration overlap, or differ at most by a factor of two, leaving the possibility that one of these time scales causally drives the other. Indeed, a previous study directly demonstrated that the temporal predictability of sensory stimuli is a critical factor in shaping alignment.^37^ Future work could dissociate the effects of trial-wise prediction and overall temporal entrainment by using much longer and highly random inter-trial intervals, such as used during a study on respiration and conditioning.^38^

In experiment 2, which manipulated trial value and task difficulty independently, we did not observe a significant effect of value on respiratory alignment and only a small effect of value on behavior. One explanation is that the overall effectiveness of the manipulation was too small to yield an effect on respiration. Other than in experiment 3, in experiment 2 only value was cued prior to each trial, whereas difficulty could only be judged during the stimulus and therefore remained unpredictable. When dealing with the independent demands imposed by value and difficulty, an optimal strategy would be to deploy only some effort toward adjusting neural resources according to the expected value and reserve some effort toward adaptively deploying resources once difficulty is known (i.e., during stimulus). Such a division of resources could lead to weaker respiratory alignment for trial value compared to an experiment involving the joint manipulation of difficulty and value, which is directly consistent with our observations. Together our data hence suggest that the alignment of respiration is driven by the anticipated effort required to successfully complete the trial. Future studies could follow this line of thought and also obtain subjective ratings of perceived effort or include (neuro-)physiological or pupil-derived signatures of effort to further corroborate a link between respiratory patterns and task-related effort.

The outcomes of perception and action are intricately linked to the physiological state of our body. Recent studies have shown that under laboratory conditions participants tend to structure their respiration systematically around expected events such as individual experimental trials. We here show that the strength of this alignment is specific to the value attributed to individual trials or the expected effort required. Hence, we tend to actively structure our respiration in a selective manner for individual upcoming required actions.

### Limitations of the study

While we observed significant differences in respiratory alignment in two experiments that were accompanied by meaningful effect sizes, these respiratory effects did not correlate significantly with parallel effects in behavior across participants. While this does not invalidate our main arguments, it calls for future studies to link trial-wise adjustments of respiration to trial-wise behavior in more detail. Also, in experiment 3 the effort-difficulty manipulation only affected reaction times but not response accuracy. We can only speculate about why this was the case. One explanation might be the limitation of participants overall accuracy by the nature of stimuli, such as their duration. Whether both the accuracy by which we perform a task and response speed are related to respiration remains a topic of ongoing research. Comparative analyses across multiple datasets have pointed to stronger effects on response speed,^2^^,^^5^ but in a recent neuroimaging study we found that the respiratory phase directly modulates EEG signatures of task relevant information that are predictive of both response times and accuracy.^28^ While accuracy primarily reflects the precision with which sensory information is encoded and utilized, reaction times are related to the speed of neural computations as well as motor executions. Future studies need to more clearly differentiate how respiration relates to each of them. Also, we note that we did not collect demographical data, and our study cannot test any association of sex or age on any of the reported effects.

Another yet unresolved question concerns whether the alignment of respiration to the experiment trials is driven by the temporal expectation of a specific stimulus, by the expectation to perform a specific action or the action itself.^5^ In principle, respiratory alignment could simply be driven by the expectation of something to happen, i.e., a stimulus to appear. However, in line with the notion of active sampling, we propose that this alignment would be stronger in the context of specific task requirements, hence the need for a motor response. On the time scales of the experiments performed here or in previous work, these three factors are all highly correlated. The stimulus-response delays are short compared to the duration of respiratory cycles, and all trials required an active response. Hence, the current data cannot dissociate these factors. To better dissociate the potential drivers of the alignment, we need experiments with clearly expected and unexpected stimuli, as well as manipulations of the stimulus-response contingencies. One possibility would be to use a go/no-go paradigm, where a stimulus is reliably expected on every trial but a motor response is required on only a subset of them.

Along this line, one should interpret the timing of the reported condition differences in respiratory alignment with care. To account for the smoothness of the data and to correct for multiple comparisons long time, these are based on a cluster-based permutation method, which is often used for continuous data, such as EEG or MEG.^39^ However, such a test only allows inference on whether differences between conditions are significant, but does not allow interpreting the time point of significant effects.^40^ Combined with the smoothness of the respiratory signal, this makes interpreting the time course in the context of causal relations, e.g., of whether effects emerge before or shortly after stimulus onset, difficult and a further topic for future work.

A last question pertains to the role of arousal in mediating the behavioral benefits of respiratory alignment. Previous studies suggest that pupil size, a marker of arousal and the activity of the noradrenergic system, varies systematically along the respiratory cycle.^41^^,^^42^ Hence, selective or optimal timing of the respiratory cycle could reflect the corresponding selective timing of arousal, which by its widespread influence on cortical function may well be responsible for concomitant improvements in behavioral performance. However, one may also speculate about an alternative interpretation, whereby increased arousal allows the better alignment of respiration, possibly because the respective pathways are in a more excitable state allowing quicker adaptation. Clearly, both the role of arousal (vs. more functionally specific neural processes) and the causality between these processes need to be investigated further. Of particular interest could be manipulations of the respiratory rhythm (e.g., breath holds) and the time-resolved analysis of markers of arousal (derived from eye tracking or EEG recordings) to obtain more insights about the directionality of the interactions of respiration, arousal, and their pacing by external events.

## Resource availability

### Lead contact

Further information and requests for additional resources and reagents should be directed to and will be fulfilled by the lead contact, Christoph Kayser (christoph.kayser@uni-bielefeld.de).

### Materials availability

This study did not generate new unique reagents.

### Data and code availability

• Behavioral and respiratory data are available on Mendeley, Database: https://doi.org/10.17632/pycr3r844w.1.
• Custom code for analyzing the behavioral and respiratory data and producing Figures 2, 3, and 4 are available on Mendeley, Databse: https://doi.org/10.17632/pycr3r844w.1.
• Any additional information required to reanalyze the data reported in this paper is available from the lead contact upon request.

## Acknowledgments

We would like to thank Sepideh Mirzaei for help with data collection.

## Author contributions

C.K., study design, data analysis, drafting of manuscript, revision of manuscript, and project overview; L.H., study design, data collection, data analysis, drafting of manuscript, and revision of manuscript; L.S., study design, data collection, data analysis, drafting of manuscript, and revision of manuscript. All authors have read and approved the final version of this manuscript.

## Declaration of interests

The authors declare no competing interests.

## STAR★Methods

### Key resources table

**Table:** 

REAGENT or RESOURCE	SOURCE	IDENTIFIER
**Deposited data**		

Behavioral data and custom code	Mendeley Data	https://doi.org/10.17632/pycr3r844w.1

**Software and algorithms**		

Custom code	Mendeley Data	https://doi.org/10.17632/pycr3r844w.1
MATLAB, 2017a	MathWorks	www.mathworks.com/

### Experimental model and study participant details

#### Human participants

The study was approved by the ethics committee of Bielefeld University (Approval Nr. 2024-037). Adult volunteers with self-reported normal vision and hearing participated after providing informed consent. All participants were compensated for their time. The data were collected anonymously and it is possible that some individuals participated in more than one of the experiments described below. Demographical data, including gender or ethnicity were not collected; the participant pool consisted of typical young university students. Prior to the study, our interest to investigate the relation between respiration and task performance was not mentioned explicitly and participants were instructed to “breathe through their nose as usual”, as if performing the experiments without wearing the measurement device. During the experiment we could not continuously monitor whether participants adhered to this instruction, leaving the possibility that during parts of the experiment participants were breathing orally.

### Method details

#### Experimental setup

The experiments were performed in a darkened and sound-proof booth (E: Box; Desone, Germany). Visual stimuli were presented on a computer monitor (27″ monitor; ASUS PG279Q, about 85 cm from participant’s head) with stimulus presentation controlled using the Psychophysics Toolbox (Version 3.0.14) in MATLAB (Version R2017a; The MathWorks). Participants responded using a computer keyboard.

As in our previous studies, respiration data were recorded using a temperature-sensitive resistor (Littelfuse Thermistor No. GT102B1K, Mouser electronics) that was inserted into disposable clinical masks, capturing the temperature changes resulting from the respiration-related airflow.^2^^,^^5^ The voltage drop across the thermistor was amplified and recorded via an ActiveTwo EEG system (BioSemi BV) at a sampling rate of 1000 Hz. We verified that this temperature signal directly reflects airflow by comparing this with direct measurements of flow performed using a short-latency airflow sensor (F1031V, Mass Airflow Sensor, Winsen).

#### Behavioral paradigms

We collected data in three experiments probing visual tasks that were also used in previous studies on respiration, and have proven useful to study the relation of respiration and behavior.^2^^,^^5^ Experiment 1, which manipulated response deadlines in a block-wise manner, relied on an emotion discrimination paradigm; experiments 2 and 3, which manipulated task difficulty and trial-value on a trial-by-trial basis, relied on a random dot motion discrimination paradigm (Figure 1A). All experiments involved a practice block, for experiments 2 and 3 a block to determine each participant’s perceptual threshold for the respective motion task and one or more blocks of experimental trials. For each task, participants were instructed to respond as fast and accurately as possible after stimulus onset.

##### Experiment 1

Participants discriminated the emotional expression (sad or happy) of briefly presented faces (subtending 15 × 12°, presented for ∼17 ms). Stimuli were obtained from the Dynamic FACES database^43^ and each trial presented a different image. Trials started with a fixation period indicated by the appearance of a central fixation dot that remained on the screen until stimulus onset. This pre-stimulus period lasted 1000 ms and was fixed. Inter-trial intervals were drawn from a uniform distribution between 3500 and 4000 ms. Importantly, this task was performed either in the context of a LONG response deadline, or an SHORT response deadline. In the LONG condition participants had 1.4 s time to submit their responses, in the SHORT condition they were given a reaction time (RT) deadline that was titrated for each participant based on their actual RTs in the LONG condition. The threshold was defined as the 30% percentile of the RT distribution in the LONG blocks. Participants performed four blocks of 100 trials for each condition, with the first half of the experiment presenting the LONG condition and the second half the short condition (hence 800 trials in total). If participants did not submit a response within a deadline period they received visual feedback and those trials were excluded for analysis. Overall we obtained data from *n* = 27 participants, for which we retained 643 ± 19 trials on average (mean ± s.e.m.) for the analysis.

##### Experiment 2

Participants judged the direction of motion (left- or right-wards) of visual random dot displays. Random dot displays lasted 660 ms, subtended 8 degrees of visual angle and contained 800 limited-lifetime dots (0.2° diameter, 8 frames life-time) moving at 3.5° per second. The coherence of dots (fraction of dots moving in the same direction) manipulated task difficulty. Trials could either be easy or hard, with hard trials presenting motion coherence around participants perceptual threshold and the easy condition featuring a coherence that was 3.5% higher than threshold. Prior to the actual task we determined participants perceptual thresholds (around 72% correct responses) using three interleaved one-up two-down staircases with multiplicative step-sizes. Importantly, within the experimental blocks each trial was assigned either a high or a low value. Value was manipulated independently of task difficulty, and both value and difficulty were pseudo-randomized across trials and hence not predictable based on the sequence of previous trials. However, value was cued prior to each trial and hence known while preparing for the upcoming stimulus. Participants were told that they could earn points during experiment for submitting correct responses and that there were trials with higher value (yielding 3 points) and with lower value (1 point). High value trials were less frequent (relative ratios of high and low value 1:2.33). At the end of the experiment participants were compensated for their time, but also received additional compensation based on their score (computed as their total score/100). For participants to be able to exploit the value of each trial they were presented with cue prior to each stimulus presentation: this was a 3-s counter, counting down the seconds until stimulus onset. The color of this indicated the value of the upcoming trial. Inter-trial intervals were 1700-2800 ms (uniform). Task difficulty was not predictable and could be experienced only during a trial. Participants performed 5 blocks of 132 trials (660 trials in total). We obtained data from *n* = 24 participants for which we retained 585 ± 14 trials on average for analysis.

##### Experiment 3

This experiment featured the same overall design as experiment 2. But rather than value and difficulty being independent variables, these were linked and both were hence predicted by a cue prior to stimulus onset. That is, the high value trials also had higher difficulty, and the low value trials had lower difficulty and participants were informed about this prior to the experiment. In addition, we increased the coherence difference between easy and difficult trials, presenting the high value trials around threshold and the low value (and easier) trials at 8% above perceptual threshold. We did so as the performance differences in experiment 2 between easy and hard trials proved rather small. Participants performed 5 blocks of 126 trials (630 trials in total). We obtained data from n = 23 participants for which we retained 601 ± 12 trials on average.

In contrast to the previous studies using these paradigms, we here presented trials with longer inter-trial intervals and with temporally reliable cues that indicated the expected stimulus onset. In our previous studies, the pre-stimulus (fixation) periods were pseudorandom and on the order of 400-1000 ms.^2^^,^^5^ Hence they provided only somewhat reliable temporal cues about stimulus onset. We here relied on longer but fixed fixation periods (see below) to allow the precise expectation of when a stimulus would appear. We also included longer inter-trial intervals. In previous studies, the inter-trial intervals were on the order of 1.3 s, resulting in an overall pacing of subsequent trials on the order of around 3 s, as typical for behavioral and neuroimaging paradigms probing sensory or cognitive processes (see Figure 1B in Harting et al.^5^). The experiments implemented here resulted in time intervals between subsequent stimulus onsets of 5.75 ± 0.10 s (mean ± SD) for experiment 1 and 7.58 ± 0.36 s and 7.42 ± 0.16 s for experiments 2 and 3. This timescale is longer than the typical respiratory cycle duration for most participants. These changes were introduced to allow a better alignment of respiration to the experimental trials based on temporal expectation by allowing for longer inter-trial intervals commensurate with respiration and by providing clear predictive cues.

### Quantification and statistical analysis

#### Data preprocessing

Data preprocessing and analysis was carried out in MATLAB (Version R2017a; The MathWorks). The respiratory data were separated into individual cycles based on the Hilbert transform and the detection of local peaks and throughs.^5^ We characterized atypical respiratory cycles by comparing the time course of individual cycles using their mean-squared distances. For each participant we excluded cycles with a distance larger than 3 standard deviations from the centroid distribution as atypical, as these may reflect sights of breath holds. Within each cycle we defined the respiratory phase as a linearly increasing variable from the beginning to the end of inspiration (defined as angle from 0 to pi) and subsequently as linearly increasing from the beginning to the end of expiration (defined as pi to 2∗pi). This phase variable was resampled to 20 Hz for subsequent analysis. From the overall data we removed trials for which the respiratory cycle at the time of stimulus onset was atypical and trials with reaction times shorter than 200 ms or longer than 3 s. For experiment 1 we also discarded trials without responses within the required deadline. Respiratory example data from one participant are shown in Figure 1B.

#### Analysis of respiratory cycle durations

For each trial we determined the respiratory cycle including stimulus onset time and calculated the duration of this cycle. We then contrasted the trial-averaged durations between conditions at the group-level using paired t-tests. In addition, we correlated the participant-wise condition-difference in cycle duration with the condition-difference in response accuracy and reaction times using Spearman’s rank correlation. The resulting r- and *p*-values are indicated in the respective scatterplots.

#### Analysis of respiratory alignment and statistics

We quantified the alignment of respiration to the experimental paradigm using a measure of phase-locking vector strength (plvs) which we computed for time epochs of the respiratory phase around stimulus onsets. This plvs was obtained by first defining the respiratory phase as a complex-valued number. Then we averaged these complex numbers across trials and used the vector length as plvs trace.^2^^,^^26^ To contrast the actual group-mean plvs against the null hypothesis of no alignment at the group-level, we derived a surrogate distribution of 4000 plvs for each participant as follows^5^: for each iteration we randomly time-shifted the individual respiratory epochs relative to stimulus onset by random and independent amounts and recalculated the participant-wise phase consistency. This resulted in 4000 surrogate group-mean plvs traces. To correct for multiple comparisons over time, we extracted for each surrogate trace the maximal value a long time and then computed the 99^th^ confidence intervals of these (hence testing at *p* < 0.01), thereby correcting for multiple comparisons a long time.

Our main hypothesis concerned whether this alignment differs between conditions placing different demands or importance systematically within participants. For this we compared the group-level plvs between conditions using paired t-tests and relied on a cluster-based permutation procedure to correct for multiple comparisons over time.^39^ We shuffled the sign of the condition-difference independently for each participant and obtained the distribution of group-level differences under the null hypothesis of no difference between conditions across 4000 randomizations. We then probed for significant clusters in the actual data based on a minimal cluster size of 250 ms and using the max-sum for cluster-forming. For these tests we report *p*-values for individual clusters and the Cohen’s D effect size at the time point of largest difference. The reported sample sizes for statistical testes correspond to the number of participants included in each study. When summarizing data across participants we use means and bootstrap confidence intervals.

As a second measure of respiratory dynamics we quantified the slope by which the plvs changes around trial onset. For this we computed the difference between the minimal value in the time period of −2 to −1 s prior to stimulus onset to the maximal value in the window of +1 to +2 s. This slope reflects how quickly any change in respiratory alignment unfolds around stimulus onset, capturing the dynamics of changes in respiratory phase. We contrasted these slopes between conditions using paired t-tests. For these we report p- and t-values and Cohen’s D as effect size. Changing the time window used to define the slope, e.g., by increasing this by half a second, or shifting this in either direction by the same amount, did not alter the main conclusions. Hence, the reported results are robust to the choice of the precise time window.
